# A Personalized, Home-Based, Multidisciplinary Outpatient Clinic for Managing Breathlessness in Chronic Obstructive Pulmonary Disease: Protocol for a Single-Arm, Mixed-Methods Cohort Study

**DOI:** 10.2196/85766

**Published:** 2026-04-02

**Authors:** Jonathan Shung-Yi Lee, Belinda Cochrane, Tracy A Smith, Sheree M Smith, Soo Foo, Luisa Garcia, Teresa Kemp

**Affiliations:** 1 Department of Respiratory Medicine Camden and Campbelltown Hospitals Sydney Australia; 2 School of Medicine Western Sydney University Sydney Australia; 3 Westmead Clinical School Faculty of Medicine and Health The University of Sydney Sydney Australia; 4 Department of Respiratory and Sleep Medicine Westmead Hospital Sydney, New South Wales Australia; 5 School of Nursing and Midwifery College of Health Adelaide University Adelaide, South Australia Australia; 6 School of Nursing and Midwifery Western Sydney University Sydney Australia

**Keywords:** chronic obstructive pulmonary disease, COPD, breathlessness, multidisciplinary, chronic disease management, breathlessness intervention service

## Abstract

**Background:**

Breathlessness is a distressing symptom for people with chronic obstructive pulmonary disease (COPD), impacting independence and community participation, resulting in reduced quality of life. With optimized treatment, nonpharmaceutical interventions deployed in a home-based breathlessness intervention service may reduce breathlessness to a manageable level.

**Objective:**

The aim of this study is to investigate whether patients with COPD experience reduced burden of chronic breathlessness and health care use following participation in the Macarthur Breathless Clinic (MBC), a multidisciplinary home-based health service intervention and to explore participant and carer experiences.

**Methods:**

The proposed research is a single-site, mixed methods, single-arm cohort study evaluating MBC. Eligible participants will have chronic breathlessness as defined by a modified Medical Research Council score of at least 2 and COPD confirmed by spirometry. Initially, participants will undergo a holistic medical assessment to understand factors contributing to breathlessness and to optimize therapy. The MBC intervention will be a 9-week, home-based, multidisciplinary program (with contributions from medical, nursing, physiotherapy, and occupational therapy clinicians). Central to this program will be coaching for breathlessness self-management using individualized, multicomponent strategies. Breathlessness impact will be assessed using questionnaire instruments measuring breathlessness mastery, symptom burden, quality of life, and psychological distress, with the Chronic Respiratory Questionnaire’s mastery subscale as primary outcome. Assessments will be undertaken at baseline, 9 weeks, and 52 weeks; 12-month exacerbation rates, health care use, and rescue medication use will be monitored and measured against participants’ historical data. Participant and carer experiences will be evaluated using qualitative methods to inform development of the service.

**Results:**

This project was funded in stages according to defined milestones, with the final milestone payment received in October 2025. Data collection commenced in March 2022. On submission of this manuscript, recruitment is complete with 92 participants having completed the protocol by March 2024. Data analysis is ongoing, and results are expected to be reported by early 2027.

**Conclusions:**

This research protocol is the first to investigate long-term outcomes following a health service intervention targeting chronic breathlessness for patients with COPD. Positive results may see breathlessness strategies from MBC or other similar health service interventions incorporated into existing models of chronic COPD care.

**Trial Registration:**

Australian and New Zealand Clinical Trial Registry ACTRN12620001330932; https://tinyurl.com/35fec8h4

**International Registered Report Identifier (IRRID):**

DERR1-10.2196/85766

## Introduction

Chronic obstructive pulmonary disease (COPD) is a multisystem disease, primarily affecting the lungs and characterized by incompletely reversible airflow obstruction [1]. COPD is common, affecting 10% of adults worldwide and 14% of Australians aged >40 years [2]. Despite treatment according to guidelines, people with COPD experience a range of symptoms. Breathlessness is often prominent among these and may contribute to physical and psychological distress, and reduced functional status and community participation [1]. Breathlessness is responsible for a high proportion of ambulance presentations, medical admissions, and health care costs [3,4]. However, patients with refractory breathlessness and their caregivers feel that there is a lack of awareness of the symptom burden among health care providers and insufficient provision to address this burden within health care services [2,5,6].

Breathlessness intervention services have been clearly shown effective in reducing the burden of breathlessness for populations with malignant and mixed etiology disease [7]. Existing breathlessness intervention service research primarily targets participants with malignant disease, in Europe, over a 4- to 6-week period, led by clinicians whose expertise is in palliative care [7]. An Australian protocol for a single-blind, randomized, parallel-group study investigating the impact of a breathlessness intervention service on people with COPD has been published; however, the study outcomes are not yet fully reported [8].

The primary aim of this study is to assess whether participants living with COPD will experience reduced breathlessness impact following participation in a bespoke 9-week intervention, the Macarthur Breathless Clinic (MBC), focused on nonpharmacological interventions such as breathing retraining, energy conservation, physical activity, and use of a handheld electric fan. A secondary aim is to monitor participants’ exacerbations, health care use, and rescue medication use after the intervention for comparison with past patterns of disease activity. Furthermore, we will use qualitative methods to assess patient and caregiver experiences.

The hypothesis is that patients with COPD will experience (1) reduced impact of breathlessness (in terms of coping ability, symptom burden, quality of life, and psychological distress); (2) reduced COPD-related health care use; and (3) report a positive consumer experience following participation in the MBC.

## Methods

### Study Design

This research is a prospective, single-arm cohort study of MBC, a breathlessness intervention service, assessed using mixed methods. The intervention is designed to adapt to incorporate components found to work well and to discard or revise those found to be less effective, informed by the experience of conducting the program.

**Figure 1:**
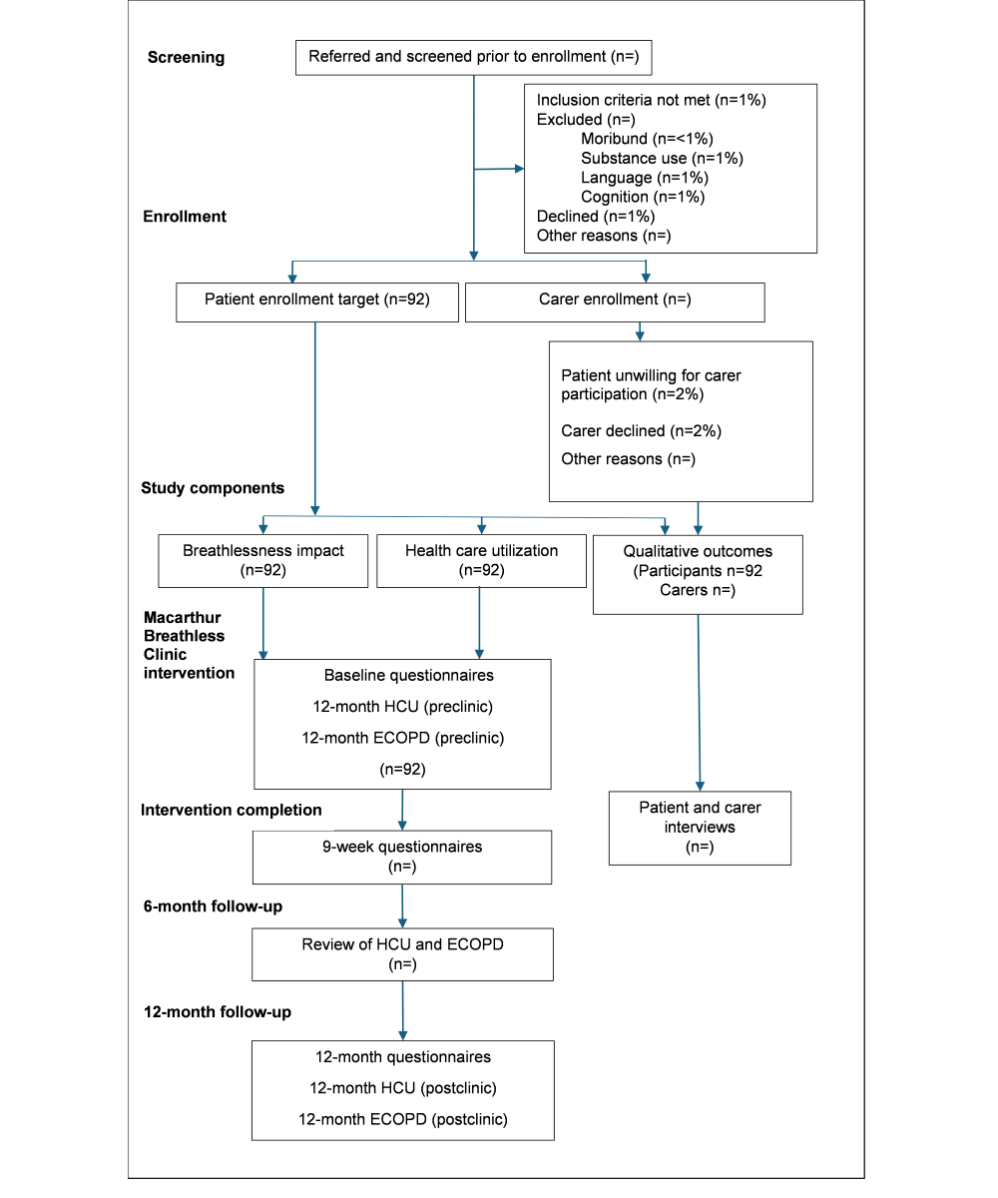
Study design. Allocated percentages are estimates only. ECOPD: exacerbation of chronic obstructive pulmonary disease; HCU: health care use.

Quantitative outcomes will be evaluated with participants acting as their own controls, before and after the clinic intervention. A 12-month follow-up with sequential cross-sectional assessments will provide an opportunity to assess breathlessness impact, exacerbations, health care use, and durability of outcomes. Qualitative outcomes for patients and their carers, if applicable, will be sought via an interviewer-administered survey in the months following completion of the clinic intervention. A flow diagram overview of the study design is shown in Figure 1.

### Ethical Considerations

This study protocol has been approved by the South Western Sydney Local Health District’s Human Research Ethics Committee on December 1, 2020, and is listed on the Australian and New Zealand Clinical Trial Registry (ACTRN12620001330932). The research will be conducted in accordance with the principles of the World Medical Association Declaration of Helsinki, ensuring participant safety, data confidentiality, and adherence to all ethical guidelines. Written informed consent will be obtained from each participant after they have been fully advised of the study aims, methods, potential risk and benefits, their right to withdraw at any time without reprisal, and the measures to protect their privacy and confidentiality. Participation will be entirely voluntary, and no monetary compensation, gifts, or incentives will be provided to participants in exchange for their time or information.

### Setting

The study will be conducted predominantly within participants’ homes across the Campbelltown, Camden, and Wollondilly local government areas in Sydney, Australia. This is an area on the fringe of metropolitan Sydney that accommodates a population with ethnic and linguistic diversity, social disadvantage, and a comparatively high proportion of Aboriginal Australians. In addition to home visits, a maximum of 2 scheduled health care interactions will occur in selected outpatient sites within the Macarthur Health Service.

### Recruitment

Clinician referrals will be sought via outpatient services or after stabilization following hospital admission for COPD-related illness. Referrals will be screened by research personnel to ensure eligibility. Previous piloting of this mode of referral and recruitment has demonstrated feasibility for achieving the recruitment target over 12 months.

Where applicable and with patient agreement, carers will be invited to participate in the trial to contribute qualitative data to reflect on their support person role and perceptions of the service.

### Sample Size

Recruitment target estimation is constrained by limited precedent data, most of which originate from research undertaken in the United Kingdom, studying participant populations, interventions, and outcomes that differ to ours [7,8]. However, pilot data from Westmead Hospital’s Breathlessness Clinic for patients with COPD (similar target population to that proposed in this study) demonstrated statistically significant and clinically relevant outcome improvements in preliminary analysis of only 10 participants [9]. The currently proposed model has been informed by their prior experience. To allow for the substantial losses to follow-up commonly seen in clinical trials in moderate to severe patients with COPD, we have set our recruitment goal at 92 participants, in anticipation of 30% (n=31) discontinuation or dropout.

### Data Collection Methods

All participant clinical and demographic data, as well as baseline and subsequent outcome data, will be collected by nonclinician MBC staff to minimize social acceptance bias. To ensure consistency in data evaluation, the same staff members will, where possible, collect outcome data for individual patients throughout the study.

A combination of outcomes has been chosen to comprehensively assess the intervention. Breathlessness impact will be measured using standardized questionnaires. These questionnaires will be administered in a pen-and-paper format in the home setting by interviewers without detailed knowledge of the intervention to further reduce bias. Participants will attempt questionnaire responses uninformed about their previous results but can be prompted with this information, if needed, in the event that the participant is otherwise unable to respond.

Health care use and exacerbation data will be gathered through structured interviews with participants. These interviews will be designed to capture detailed information about health care visits, hospitalizations, and other relevant health care interactions over the study period. The self-reported data from these interviews will be systematically cross-referenced with participants’ health records to improve accuracy.

Qualitative data will be collected through in-depth interviews using a predefined semistructured interview script for both participants and carers. After obtaining permission, interviews will be recorded and transcribed verbatim for subsequent analysis. After transcription, recordings will be destroyed, and transcripts will be stored in the participant research files.

The purpose of surveying participant and carer experiences is partly a pragmatic attempt to inform development and adaptation of the clinical intervention to better suit end users and should also provide a platform for future research. The interviews will aim to capture participants’ experiences and perceptions of the intervention, providing valuable insights about any impact on their daily lives. Similarly, for the carers, we hope to explore their perception of how the intervention affects their care recipient’s coping ability and whether the caregiver notices any change in the burden of care.

The semistructured interview transcript can be found in Multimedia Appendix 1.

### Inclusion and Exclusion Criteria

The proposed participant eligibility criteria are shown in Textbox 1.

**Participant eligibility criteria.**

**Inclusion criteria**

- Clinical and spirometric diagnosis of moderate to severe chronic obstructive pulmonary disease (COPD) with forced expiratory volume in 1 second (FEV1) <80% predicted and FEV1/forced vital capacity ratio <70%
- Severe breathlessness with modified Medical Research Council score ≥2
- Aged ≥40 years

**Exclusion criteria**

- Bed-bound or moribund
- Cognitive impairment that precludes ability to engage and participate in education strategies
- Current active diagnosis of cancer, substance abuse (other than nicotine dependence), or other uncontrolled medical or psychiatric disorder
- Moderate or severe exacerbation of COPD (defined as an escalation of treatment to include systemic corticosteroids and/or antibiotics [10]) within 4 weeks
- Insufficient command of the English language to comprehend and engage with interventions
- Current enrollment in pulmonary rehabilitation program (may participate if still eligible upon program completion or if involved in a long-term maintenance program)

Description of Intervention: MBC

The proposed intervention, the MBC, is a 9-week program to manage chronic refractory breathlessness. The MBC team will include a respiratory physician, occupational therapist, specialist respiratory nurse, and physiotherapist with referrals as needed for dietetic and speech pathology services. The MBC itself will comprise 9 health care interactions of 60 to 90 minutes, with most visits occurring within the patient’s home as outlined in Table 1.

**Table 1:** Macarthur Breathless Clinic 9-week program.

Schedule	Location	Activity
Week 1	Patient’s home	Initial assessment, respiratory nurse
Week 2	Outpatient clinic	Medical assessment by respiratory physician Multidisciplinary team assessment of breathlessness, contributing factors and impacts to develop recommendations and management plan Introduction to nonpharmacologic strategies, including provision and use of a battery-operated handheld fan Fact sheets on managing breathlessness
Week 3	Patient’s home	Physiotherapist
Week 4	Patient’s home	Occupational therapist
Week 5	Patient’s home	Respiratory nurse
Week 6	Patient’s home	Physiotherapist
Week 7	Patient’s home	Respiratory nurse
Week 8	Patient’s home	Final assessments, research assistant
Week 9	Outpatient clinic	Closure and follow-up arrangements, multidisciplinary team

An initial holistic medical assessment will be performed by an experienced multidisciplinary team, including a respiratory physician, specialist respiratory nurse, and respiratory physiotherapist. The holistic assessment framework that will be used for breathlessness is systematic and encompasses the skill set of the multidisciplinary team ensuring the whole person is considered. The assessment template in Multimedia Appendix 2 outlines the domains considered. The assessment recognizes the multifactorial nature of breathlessness, with possible contributions from many interconnected parts, including medical history, comorbid diseases, medications, mental and social factors, understanding and beliefs about breathlessness, self-management strategies, inhaler technique, current functional capacity, exercise history, and even incontinence.

Following initial assessment and optimization of medications, nonpharmacological therapies will commence. Through the MBC, patients and carers will receive education about breathlessness management and daily coping strategies to enhance independence and productivity. The clinical team will coach participants through individualized strategies to improve management of breathlessness in daily activities (Table 2) based on Cambridge Breathlessness Intervention Service’s “breathing, thinking, functioning” clinical model [11]. A broad repertoire of standard interventions will be tailored to individual patients’ needs, goals, and preferences.

**Table 2:** Nonpharmacologic breathlessness interventions.

Theme	Examples
Beliefs	Validation of breathlessness Clarifying mistaken beliefs—for example, breathlessness is not always due to low oxygen levels and breathlessness itself is not dangerous Motivational interviewing
Strategies	Use of a handheld battery-operated fan Breathing techniques such as “breathing around a rectangle,” “pursed lip breathing,” “flickering the candle,” “paced breathing,” or “diaphragmatic breathing” explained with an accompanying fact sheet Positions to relieve breathlessness Sputum clearance techniques
Exercise	Walking program with use of a pedometer Strengthening exercises
Aids/efficiency	Shower chair 4-wheel walker Energy conservation Home modifications Referral to dietitian if underweight or malnourished
Education and self-management	Inhaler technique Disease education (including medications and other therapies) Written breathlessness action plans Written chronic obstructive pulmonary disease exacerbation action plan Breathlessness DVD to reinforce key interventions Relaxation CD Fact sheets on managing breathlessness
Miscellaneous	Carer support Assessment and management of incontinence

**Figure 2:**
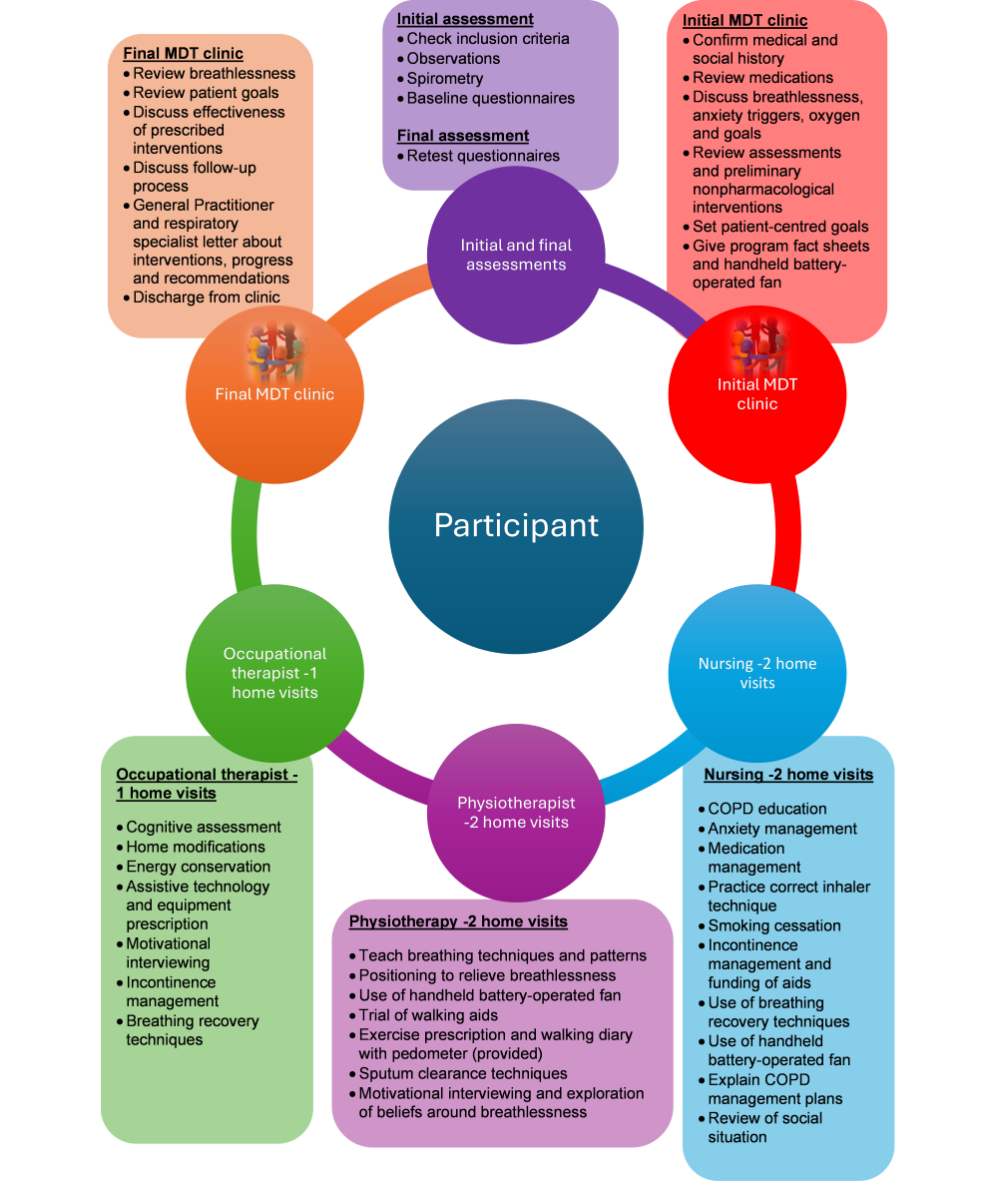
Clinician roles and coaching topics. COPD: chronic obstructive pulmonary disease; MDT: multidisciplinary team.

The role of each clinician and coaching topics covered is illustrated in Figure 2, noting that any cross-over of topics between clinicians will serve as reinforcement of strategies provided or will allow colleague cross-cover.

There will be fortnightly multidisciplinary team case conferencing of all participants during their 9-week intervention. Case conference discussion will aim to review and/or revise patient goals and optimize, coordinate, troubleshoot, and plan intervention activities. At the patient’s final outpatient clinic review, the multidisciplinary team will revisit patient goals, discuss next steps, and seek informal feedback on the program.

### Outcome Measures

#### Breathlessness Outcomes

The proposed primary outcome is mastery of breathlessness, as measured by the Chronic Respiratory Questionnaire (CRQ) subscale of mastery [12].

The proposed secondary outcomes, described below, will measure the impact of the intervention using several scales to provide a full understanding of change across a number of dimensions.

The CRQ is a validated measure of quality of life used in chronic lung diseases, with a particular emphasis on breathlessness. It consists of 4 subscales: dyspnoea, fatigue, emotion, and mastery. In each scale, higher scores reflect better health. The minimal clinically important difference (MCID) for each subscale individually is an increase of 0.5 on a 7-point scale, with a change of 1 representing a moderate change and 1.5 a large change [12].

For breathlessness intensity, unpleasantness, and confidence numerical rating scales, international guidelines recommend assessing both intensity and unpleasantness of breathlessness [13]. The MCID for these measures is a change of 1 unit [14].

The COPD Assessment Test (CAT) is a validated 8 item tool that provides a reliable measure of the impact of COPD on a patient’s health status. Higher scores reflect worse health with an MCID of 2 [15].

The Hospital Anxiety Depression Scale is a 14 item self-reported measure with anxiety and depression subscales each with 7 items. An MCID of 1.5 points for Hospital Anxiety Depression Scale in COPD populations has been suggested [16].

The EQ-5D-5L is a standardized measure of health status that provides a simple, generic measure of health for clinical and economic appraisal. The scale explores the degree of difficulty across 5 domains: mobility, self-care, usual activities, pain or discomfort, and anxiety or depression. A weighted index value will be derived, ranging between −0.59 and 1, which can be converted to quality-adjusted life years based on the Australian values set. Higher scores reflect better health, and the mean MCID is 0.051. It also includes a visual analog scale 0 to 100, rating overall health from worst to best with a mean minimum important difference of 6.9 (range 6.5-8.0) [17].

Pedometer readings of average number of steps per day over the past 7 days will be recorded. There is no consensus regarding the MCID for this measure.

The Dalhousie Dyspnoea and Perceived Exertion Scale tracks dyspnea and perceived exertion. The scale consists of 7 pictures representing breathing effort, chest tightness, throat closure, and leg effort, with increasing severity depicted in the pictures, traversing from left to right [18].

#### Health Care Resource Use Outcomes

Self-reported exacerbation, emergency department (ED) visit, and hospitalization data will be collected. South Western Sydney Local Health District’s electronic medical records will also be systematically reviewed to verify participant self-reports. Data will be updated at 6 and 12 months from MBC enrollment and will include the annual number of the following:

- ED presentations (all presentations), lasting <24 hours, that do not result in hospitalization, as well as breathlessness-related ED presentations, lasting <24 hours, that do not result in hospitalization
- Unplanned hospitalizations, including unplanned hospitalizations due to breathlessness
- Hospital length of stay in bed days (all unplanned hospitalizations), as well as breathlessness-related hospital bed days
- Exacerbations of COPD counted by patient-reported activation of COPD action plan, along with occasions of prescription of antibiotics and/or corticosteroid for the treatment of symptoms of respiratory exacerbation during hospitalized events

#### Statistical Analysis Plan

Descriptive statistics will be applied to the study population to characterize them in terms of demographic and baseline clinical parameters. Normally distributed continuous variables will be expressed as mean (SD), nonparametric variables will be defined by median and range, while categorical variables will be expressed as percentages.

Breathlessness outcomes will be compared across 3 time points: baseline, 9 weeks, and 52 weeks using repeated measures ANOVA. Predictors of response will be sought using linear regression models with sex, forced expiratory volume (FEV) in 1 second, ratio of FEV to forced vital capacity, BMI, Montreal Cognitive Assessment, and baseline anxiety and depression scores as prespecified variables. Health care use measures and exacerbation data for the 12 months prior to MBC enrollment will be compared with the corresponding values for the 12 months after MBC enrollment, using Student 2-tailed *t* tests or nonparametric tests as appropriate. Protocol completer and dropout populations will be compared in terms of baseline characteristics (sex, age, FEV in 1 second, pack years, BMI, CRQ-Mastery subscale, CAT score, and Montreal Cognitive Assessment) to investigate potential selection bias.

Finally, comparison will be made with patients’ baseline data from the year prior to enrollment in the breathless clinic, benchmarked against nonparticipant comparator hospital deidentified patient medical records with (1) ED presentations for reasons of breathlessness, triaged as “shortness of breath—respiratory” or (2) hospitalized with exacerbations of COPD or heart failure as per diagnostic related group assignment. These benchmarking comparisons will use deidentified electronic medical records and will contribute to exploratory analyses undertaken to better understand the health care use of our sample cohort in the context of the overall health care use patterns of our local population “breathless” groups.

#### Qualitative Outcomes

Patients and carers (where relevant) will undertake semistructured, audio-recorded interviews up to 9 weeks after completion of MBC. These interviews will explore participant perceptions of the MBC, any particularly helpful components, and seek recommendations for future versions of the service (Multimedia Appendix 1). To identify key concepts and themes, thematic analysis will be conducted [19]. Interviews will be coded by 2 researchers, one of whom has been involved in the conduct of the MBC and a researcher from the Westmead Breathlessness Service, to provide comprehensive assessment of the data. Input from additional researchers, experienced in qualitative methods, will provide triangulation of the results to enhance trustworthiness of the analysis. The qualitative data will complement the quantitative findings and offer a more complete understanding of the intervention’s effects.

## Results

Data collection commenced in March 2022, and recruitment is now complete, with 92 participants finishing the protocol by the end of March 2024. Data analysis is ongoing, and results are anticipated in early 2027. This project has been funded incrementally according to achievement of predefined milestones. The final milestone payment was received in October 2025.

## Discussion

### Anticipated Findings

This study adds to the literature by evaluating the change from baseline after a 9-week multidisciplinary, home-based health intervention to address the symptom of breathlessness in a cohort of Australian patients who have moderate to severe COPD. The study based on this protocol will provide important insights into applying this type of intervention to patients with COPD, a chronic disease. The authors anticipate that this study will help to inform models of care for patients with COPD enduring breathlessness as a persistent and distressing symptom, providing information about the potential durability of these interventions, and the characteristics of patients with COPD who might be most likely to benefit.

### Comparison With Prior Work

Breathlessness intervention services have been shown to be effective for breathlessness over the short term [7] The first examples of these services targeted patients with malignant disease, using complex, multicomponent, nonpharmacological interventions for the management of breathlessness [20]. Subsequent trials enrolled heterogeneous populations of patients with a mixture of malignant and nonmalignant diseases, including some patients with COPD. The authors are aware of a recently completed randomized controlled trial in Australia targeting a population of patients with COPD using a breathlessness intervention model similar to our own. Breathlessness in COPD often remains persistent and distressing despite best pharmacological treatments. However, COPD is a chronic disease, and so patients may live for many years with this burden. For these patients, not only are effective treatment strategies needed but benefit must be sustained over the long term. The proposed protocol uses a multidisciplinary home-based complex health service intervention similar in type to those known to confer significant short-term benefits for breathless patients in mixed disease populations, aiming to confirm its applicability to breathless patients with COPD, and also exploring the durability of benefits and best selection of patients.

### Strengths and Limitations

The study’s focus on a homogeneous disease population of patients with moderate to severe COPD will ensure that the results are highly relevant to this specific group. This targeted approach will allow for more precise conclusions regarding the usefulness in this cohort of the intervention being tested. That said, we will recruit a wide range of patients with COPD in terms of severity and exacerbation susceptibility, ensuring broad applicability to this important, and large, patient group. Thus, our results will complement the existing evidence base, as most previously published data include more heterogeneous participant populations; patients with malignant disease, and/or patients with a variety of restrictive and obstructive nonmalignant respiratory disease.

To our knowledge this is the first long-term study protocol for this type of intervention service. By spanning a longer period, an entire year, the study will mitigate the potential confounding effects of seasonal variations in COPD symptoms and exacerbations. This will ensure that the observed outcomes are more likely attributable to the intervention rather than external seasonal factors. Additionally, the proposed intervention requires behavior change, which can take time to implement. By having a longer duration than previous interventions, we hope to enable patients to embed into their routine the suggested nonpharmacological strategies and to thus realize a durable effect beyond the 9 weeks of the program.

The 12-month follow-up period will also provide insight into the durability of the intervention response over a prolonged period. To date, the scientific literature contains few reports of outcomes for this type of intervention extending beyond 2 or 3 months. Showing sustained symptomatic benefits for this type of population with nonmalignant, chronic disease is perhaps much more important than demonstrating a short-term response.

Finally, the use of the CAT as a key assessment tool will underscore the study’s relevance to clinical practice. The CAT is a validated, disease-specific instrument that provides a comprehensive evaluation of COPD symptoms and their impact on patients’ quality of life. This may allow for the MBC to be ranked against other treatment interventions used in COPD, so that any positive study findings can be integrated into existing treatment paradigms, thereby facilitating the translation of research into practice.

However, this study has several limitations that must be acknowledged. First and foremost is the nonrandomized study design; all recruited participants will receive the intervention and act as their own controls. This design could introduce both time and attrition bias and is a clear limitation on the level of evidence produced. The authors were aware of a randomized controlled trial [8] underway in another Australian center using a similar model to our protocol, and we therefore chose a nonrandomized study design to address clinical need while still demonstrating translatability to a different population and setting. Given that COPD is a chronic and progressive disease, evaluating the impact of breathlessness over time may still yield valuable insights, provided the results are interpreted carefully. While potential mortality among participants with more severe disease may skew study results, on the other hand, worsening disease trajectory may mask benefits when determining results over the longer term. Considering this, even if patients appear to be stable with respect to outcome measures at 12 months, this may actually represent improvement compared with the expected trajectory in COPD, which is one of deterioration. In fact, clinically significant breathlessness is a known marker of poor prognosis for patients with COPD [21], and so our target population will be expected to deteriorate more rapidly than the COPD group as a whole.

The study’s proposed setting, a single metropolitan center situated within a large semirural area, may limit the general applicability of our results. However, the authors are aware of concurrent research on this topic being conducted at other centers and health districts throughout Australia, which should help to address this.

Our study protocol excludes patients based on English language proficiency, which represents another important limitation. Ideally language should not prevent equitable access to medical care nor opportunities to participate in health research. The decision to include only English-speaking participants was made due to significant ethnic and cultural differences in the expression and description of breathlessness. Additionally, some of the outcome measures are only validated in English. The exclusion of people who do not speak English will limit the generalizability of our results to the English-speaking population. The authors estimate the impact of exclusion due to English proficiency to be small as shown in Figure 1, noting that exclusion percentages are estimates based on census data. The 2021 census data from the Australian Bureau of Statistics inform that the Macarthur and Wollondilly local government areas have, when compared with Sydney and New South Wales, a higher concentration of English-only speakers [22]. To mitigate the impact on generalizability and perhaps to provide an alternative means to characterize and express the severity of breathlessness in future iterations of the program, the Dalhousie pictorial scales, which help bypass literacy and language barriers, have been included as an outcome measure.

Finally, regarding COPD exacerbations, the authors recognize that patient recall of these events is notoriously poor, particularly for milder events, either self-treated or treated via primary care in the community. Moreover, Australia’s national guidelines, COPD-X, recommend that such patients have an exacerbation plan, along with prescriptions for relevant rescue medications making it even more complicated to establish the timing of these events. We will seek to confirm more severe events, resulting in an ED visit or hospitalization, systematically using local hospital electronic medical records. However, patient reports of exacerbation and health care use events will be sought nonetheless, given the possibility of capturing additional events occurring outside our health district, interstate, or within primary care, while acknowledging that the reporting and therefore capture of these events will be less reliable.

### Future Directions

Breathlessness intervention services are now established as effective models to reduce the burden of breathlessness in cohorts with both malignant and nonmalignant disease. However, questions remain about which components are most important (clinician expertise or strategy type), durability of benefits, patient selection, and the most cost-effective models. While we have preliminary answers, future research should aim to fine-tune these interventions to provide the best breathlessness solutions for both patients and health systems.

### Conclusions

The proposed study will be an important addition to the growing body of evidence about the use of nonpharmacological interventions for chronic refractory breathlessness in patients with COPD. Positive results would support incorporation of strategies from breathlessness intervention services, such as MBC into chronic disease models for COPD care, with potential to lighten the burden of chronic breathlessness for patients with COPD.

## Appendix

Multimedia Appendix 1 Qualitative outcomes—semistructured interview questions.

Multimedia Appendix 2 Initial assessment template—Macarthur Breathless Clinic.

## Abbreviations

CAT: COPD Assessment Test
COPD: chronic obstructive pulmonary disease
CRQ: Chronic Respiratory Questionnaire
ED: emergency department
FEV: forced expiratory volume
MBC: Macarthur Breathless Clinic
MCID: minimal clinically important difference
