# Supplementary material for: A Personalized, Home-Based, Multidisciplinary Outpatient Clinic for Managing Breathlessness in Chronic Obstructive Pulmonary Disease: Protocol for a Single-Arm, Mixed-Methods Cohort Study
Source: JMIR Res Protoc. 2026 Apr 2;15:e85766. doi: 10.2196/85766 (PMC13087554; doi:10.2196/85766)
Supplement: Multimedia Appendix 1 [file resprot_v15i1e85766_app1.docx]

**Appendix 1: Qualitative Outcomes - Interview Questions**

Consumer Interview – Patient

**Breathlessness**

1. How (if at all) did BC affect the severity of your breathlessness?
2. How (if at all) did BC affect the unpleasantness of your breathlessness?
3. After completing BC does your breathlessness bother you less or do you think that you manage it better?
4. Has your confidence to manage breathlessness in public changed? How? Please give examples.
5. Prior to BC, were you anxious about your breathing or did you feel down in the dumps about it? Since completing BC has this changed in any way?
6. If you have a partner/carer - during an attack of breathlessness, do you find that they are more able to support you? How?

**Activity**

1. How (if at all) did BC affect your ability to get around the house or venture out?
2. How has BC affected your ability to cope at home or undertake daily household chores?
3. Since completing BC, have your social activities changed? e.g., shopping, coffee with friends, outings (How?)
4. Starting BC, did you set goals? (If yes, please explain how you went?)
5. After completing BC, has your life changed in any way? How?

**Clinic**

To remind you and assist you responding to the following questions, BC components and activities include: breathing techniques, posture, hand-held electric fan, self-talk, exercise goals, home modifications, gait aids, other devices to assist function, breathlessness plan, relaxation CD, breathlessness DVD.

1. Did participating in BC provide any unexpected experiences, effects or surprises for you?
2. Thinking of the clinic activities, which, if any, were helpful? Why or why not?
3. Do you think that the number of appointments were enough, too many, not enough?
4. Were the number of BC appointments difficult to include in your schedule?
5. What worked well in BC?
6. Is there anything that you would change about the BC to make it more helpful?

**Conclusion**

1. In retrospect, is there anything you would like to add or is there anything that you feel that the BC team should know about your experience with the BC?
2. What advice would you give other people with COPD and breathlessness about whether to enrol in the clinic and how to get the most out of it?
3. Which, if any, components of BC will you use in future or incorporate into your daily routine

Consumer Interview – Partner/Carer

You care for someone who suffers with severe breathlessness.

Since attending BC in your view:

1. Do you think that they cope better with their breathlessness? In what ways?
2. Have they increased their social activities? Please expand?
3. Has their overall level of activity changed? In what way?
4. Have they changed in terms of their stress levels or mood? In what way?
5. Do you feel more confident to support them?
6. Was managing the number of BC appointments difficult to manage?
7. Were any aspects of BC particularly helpful to you? Which?
8. Have you any recommendations that would improve BC?
9. Are there any other comments about your experience with the BC?
