# Supplementary material for: A Personalized, Home-Based, Multidisciplinary Outpatient Clinic for Managing Breathlessness in Chronic Obstructive Pulmonary Disease: Protocol for a Single-Arm, Mixed-Methods Cohort Study
Source: JMIR Res Protoc. 2026 Apr 2;15:e85766. doi: 10.2196/85766 (PMC13087554; doi:10.2196/85766)
Supplement: Multimedia Appendix 2 [file resprot_v15i1e85766_app2.docx]

**Appendix 2: Initial assessment template**

**BREATHLESS CLINIC INITIAL ASSESSMENT**

Date: Name: MRN:

Address:

Referred by:

Aboriginal or Torres Strait Islander?

If yes, registered for Close the Gap? Yes/ No

GP Name:

Names & relationships accompanying patient:

Health care professionals in attendance:

**MEDICAL ASSESSMENT**

Factors contributing to breathlessness (list):

Current medications

| Daily |  |
| --- | --- |
| BD |  |
| TDS |  |
| QID |  |
| PRN |  |
| Other |  |

Inhalers

| Reliever | ICS | LAMA | LABA | LAMA/LABA | ICS/LABA | ICS/LAMA/LABA |
| --- | --- | --- | --- | --- | --- | --- |
|  |  |  |  |  |  |  |

Respiratory History:

Smoking Hx: Pack years;

Cough Hx:

Home O2:

PAP therapy:

COPD Action plan: (yes/no, last exacerbation, exacerbations per year, allergies to antibiotics)

Haematologic:

Metabolic:

Cardiac:

Mental Health:

Exercise:

Other:

Vaccinations:

**NURSING ASSESSMENT**

| Examination | H: W: BMI: SaO2: PR: BP: |
| --- | --- |
| Investigations | Post bronchodilator spirometry:  FEV1 value________Predicted-  FVC value_________Predicted-  FEV1/FVC  Quality rating:  Imaging (if relevant):  Bloods: |

Breathing:

Thinking:

Functioning:

**PHYSIOTHERAPY ASSESSMENT**

**Occupational Therapy Assessment**

**PLAN**
